# The Association Between Gaming Practices and Scholastic Performance Among Medical Students in India: Case-Control Study

**DOI:** 10.2196/22235

**Published:** 2021-09-09

**Authors:** Deodatt Madhav Suryawanshi, Divya Rajaseharan, Raghuram Venugopal, Madhu Mathew, Anju Joy, Ramchandra Goyal

**Affiliations:** 1 Department of Community Medicine Trichy SRM Medical College Hospital and Research Centre Trichy, Tamil Nadu India; 2 Department of Psychiatry Trichy SRM Medical College Hospital and Research Centre Trichy India

**Keywords:** gaming, gaming disorder, medical students, gaming addiction, scholastic performance, academic performance, addiction, smartphones, mobile phones, youth, medical education

## Abstract

**Background:**

Gaming is a billion-dollar industry that is expanding at a compound annual growth rate of 9% to 14.3%, with the biggest market in Southeast Asian countries. The availability of low-cost smartphones and the ease at which the internet can be accessed have made gaming popular among youth, who enjoy it as a leisure activity. According to the World Health Organization, excessive indulgence in gaming can lead to gaming disorder. Medical students indulging in excessive gaming can succumb to gaming disorder, which can affect their scholastic performance.

**Objective:**

This study aimed to assess the association between gaming practices and scholastic performance among medical students.

**Methods:**

This study used a case-control design, where 448 medical undergraduate students (first year to prefinal) were preliminarily surveyed using universal sampling on their gaming practices in the last 6 months. Out of this sample, the 91 participants who admitted to gaming in the past 6 months were recruited as cases, while participants who never engaged in gaming in the last 6 months were recruited as controls. Both the cases and controls were matched for age and gender in a 1:1 ratio. The internal assessment scores (based on 2 midterms completed in the last 6 months) of cases and controls were compared. The Snedecor F test was used to determine the association between the number of hours spent gaming and internal assessment scores (%), while the Student *t* test was used to determine significant differences between the internal assessment scores of cases and controls. Odds ratios were calculated to identify the risk of poor scholastic performance among cases compared to the controls. The prevalence of gaming disorder among cases was assessed using the Gaming Addiction Scale (GAS).

**Results:**

The frequency of gaming (in hours) was not associated with mean internal assessment scores (*P*=.13). Male cases reported significantly lower internal assessment scores compared to male controls (*P*=*.*005 vs *P*=.01), whereas no significant differences were observed between the internal assessment scores of female cases and controls (*P*=.89 vs *P*=.59). A negative correlation was observed between GAS scores and internal assessment scores (*r*=–0.02). The prevalence of gaming disorder using the GAS was observed to be 6.3% (28/448) in the study population and 31% (28/91) among cases. The risk of low scores (<50%) among gamers was observed to be 1.9 (95% CI 1.04-3.44, *P*=.03) times higher in the first midterm and 1.80 (95% CI 0.97-3.36, *P*=.06) times higher in the second midterm compared to nongamers.

**Conclusions:**

The findings suggest that excessive gaming adversely affects the scholastic performance of male participants more than female participants. Awareness about gaming disorder needs to be created among students, parents, and teachers. Treatment services should be made available to medical students with gaming disorders.

## Introduction

In 2019, the global gaming market was valued at US $151.55 billion, growing at a compound annual growth rate of 9% to 14.3% and expected to reach US $256.97 billion by 2025, with the largest market in the Asia Pacific region. Of all the available gaming platforms (PC, PlayStation, Xbox), smartphones remain the most utilized gaming platform at present, earning US $64.4 billion in 2019 [1]. India also has a rapidly growing gaming market, with an annual growth rate of 14.3% valued at US $890 million currently [2]. This growth is driven by the rising younger population, higher disposable incomes, the introduction of new gaming genres, and the increasing number of smartphone and tablet users [2].

Although considered a harmless leisure activity, excessive indulgence in gaming can lead to possible internet gaming disorder [3]. In the 11th Revision of the International Classification of Diseases, the World Health Organization recognized excessive gaming as a disorder “characterized by impaired control over gaming, increasing priority given to gaming over other activities to the extent that gaming takes precedence over other interests and daily activities, and continuation or escalation of gaming despite the occurrence of negative consequences” [4].

Recent studies have documented significant impairment of physical, psychological, social, and work-related problems such as insomnia, increased irritability and aggression, depressive and/or anxiety symptoms, poor academic performance, and neglect of interpersonal relationships with excessive and problematic gaming [5-7].

The medical curriculum is vast and requires extensive reading and dedication. In such circumstances, indulgence in excessive gaming among students can lead to gaming disorder, which can affect their scholastic performance. This study aimed to shed light on whether gaming practices among medical students affect their scholastic performance. Hence, this study was conducted with the following objectives:

1. To study the amount and nature of gaming practices among medical students;
2. To assess the prevalence of gaming disorder among medical students;
3. To study the association between gaming practices and scholastic performance among medical students.

## Methods

The study attempted to demonstrate the association between gaming practices and the scholastic performance of medical students.

### Study Design and Ethical Clearance

The study used a case-control design and was conducted during the period of October and November 2019 in a medical college in the Trichy District of Tamil Nadu, India. Ethical clearance was obtained from the Institutional Ethical Committee of Trichy SRM Medical College (1007/TSRMMCH&RC/ME-1/2019-IEC no:039). Informed written consent was obtained from all the participants. If the enrolled participants were not interviewed on a specified date, they were interviewed subsequently at a time and place of their convenience. The purpose of the study was explained in detail and assured that the data collected would be used only for scientific purposes. Ethical principles such as respect for the person and confidentiality of their data were strictly adhered to.

### Recruitment of Cases and Controls

**Figure 1:**
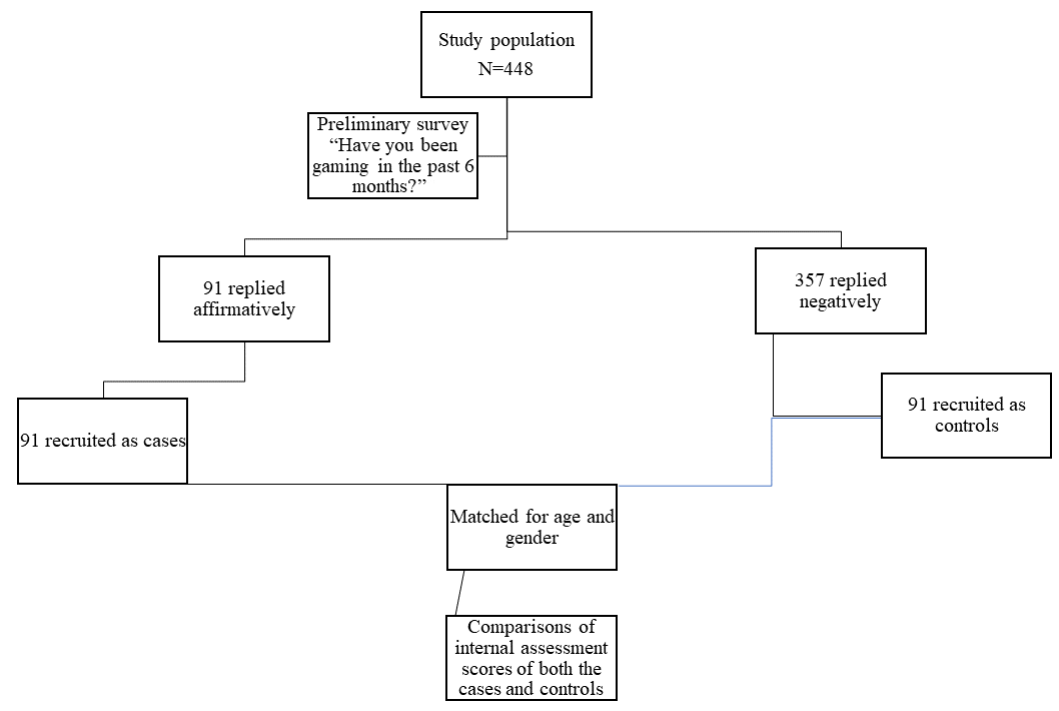
Recruitment of cases and controls.

A total of 448 undergraduate medical students in their first to prefinal year were included as participants using the universal sampling technique (Figure 1). The study preliminarily surveyed the entire study population of 448 students using personal interviews. All 448 participants were asked only 1 question: “Have you been gaming in the last 6 months?” From this preliminary sample of 448 surveyed students, 91 students replied affirmatively and were recruited as cases in the study. Following this, the investigator used purposive sampling to select 91 controls from the remaining 357 students who had never indulged in gaming in the last 6 months, and matched both cases and controls for age and gender. The controls selected were matched for age and sex using a 1:1 ratio. The frequency of gaming hours per week was assessed among the cases. The internal assessment scores of the two midterm examinations held in the last 6 months were accessed from the students’ records kept by the institution after obtaining written permission from the students and the Institutional Ethical Committee. The internal assessment scores of cases and controls were then compared and recorded in percentages.

To assess the prevalence of gaming disorder, the Gaming Addiction Scale (GAS) by Lemmens et al [8] was used. The GAS is a pretested, prevalidated scale with a Cronbach alpha of .82 to .87 [8]. It has 7 items: salience, tolerance, mood modification, relapse, withdrawal, conflict, and problems. Each item has three questions with a score range of 0 to 5 with all the components scored on a Likert scale: 1=never, 2=rarely, 3=sometimes, 4=often, and 5=very often. The investigators used the monothetic format in the study, that is, a score of >3 for all items being indicative of gaming addiction. Lemmens himself hypothesized that the monothetic format would lead to a better estimate of the prevalence of addiction than the polythetic format [8]. Therefore, the investigators used the GAS according to protocol, but for the convenience of analysis, the investigators summed up the total score of all 7 items, and classified participants with a score of ≥63 as having a gaming disorder.

### Statistical Analysis

The data entry and analysis were done using SPSS software (version 21, IBM Corp). Descriptive statistics were used for analyzing sociodemographic details, frequency, and type of gaming. The Snedecor F test and the Student *t* test were used to determine the association between the hours spent gaming and scholastic performance, and gaming and internal assessment scores, respectively. Odds ratios were used to calculate the risk of low internal assessment scores among cases and controls. The correlation coefficient (*r*) was used to determine the correlation between the GAS scores and internal assessment scores.

## Results

Of the 448 students who were preliminarily surveyed, 91 were allocated as cases and 91 as controls**.** Out of the 91 cases, 49 (53.8%) were female and 42 (46.2%) were male. The majority of cases (80/91, 87.9%) were aged 19 to 23 years. In terms of gaming platform, 87 (95.6%) used a mobile phone, 3 (3.4%) used a personal computer or laptop, and 1 (1.0%) used Xbox (Table 1).

**Table 1:** Age distribution and gaming characteristics of cases (n=91).

Characteristic			Female, n (%)		Male, n (%)	Total, n (%)		*P* value	
**Age (years)**									.57
	≤18	7 (7.6)		3 (3.3)		10 (11.1)			
	19-23	42 (46)		38 (41.7)		80 (87.9)			
	≥24	0 (0)		1 (1.1)		1 (1.0)			
**Gaming platform used**									.75
	Mobile phone	48 (52.7)		39 (42.8)		87 (95.6)			
	Mobile phone/PC	1 (1.1)		2 (2.1)		3 (3.4)			
	Xbox	0 (0)		1 (1.1)		1 (1.0)			
**Hours per week spent gaming**									.47
	≤10.0	12 (13.1)		16 (17.5)		28 (30.8)			
	10.1-25.0	28 (30.7)		22 (24.1)		50 (55.0)			
	25.1-40.0	5 (5.4)		2 (2.1)		7 (7.6)			
	40.1-55.0	3 (3.2)		0 (0)		3 (3.2)			
	≥55.1	1 (1.1)		2 (2.1)		3 (3.2)			
**Gaming Addiction Scale score**									.97
	<63.0	34 (37.3)		29 (31.8)		63 (69.2)			
	≥63.0	15 (16.4)		13 (14.2)		28 (30.7)			

The frequency of playing games was assessed for a typical working day in hours and then calculated for a 7-day week. In this study, more than half of the cases (50/91, 55.0%) spent 10-25 hours per week gaming, 28 (30.8%) cases spent less than 10 hours per week, and 6 (6.4%) cases spent more than 40 hours per week (Table 1). There was no significant difference observed in the internal assessment scores of those who played games for more hours than those who played for fewer hours (*P*=.13).

Mean scores among cases were 5.2% lower compared to the controls (mean score 48.7 vs 53.9, *P*=.01) in the first internal assessment and 4.1% lower (mean score 50.2 vs 54.3, *P*=.01) in the second internal assessment.

**Figure 2:**
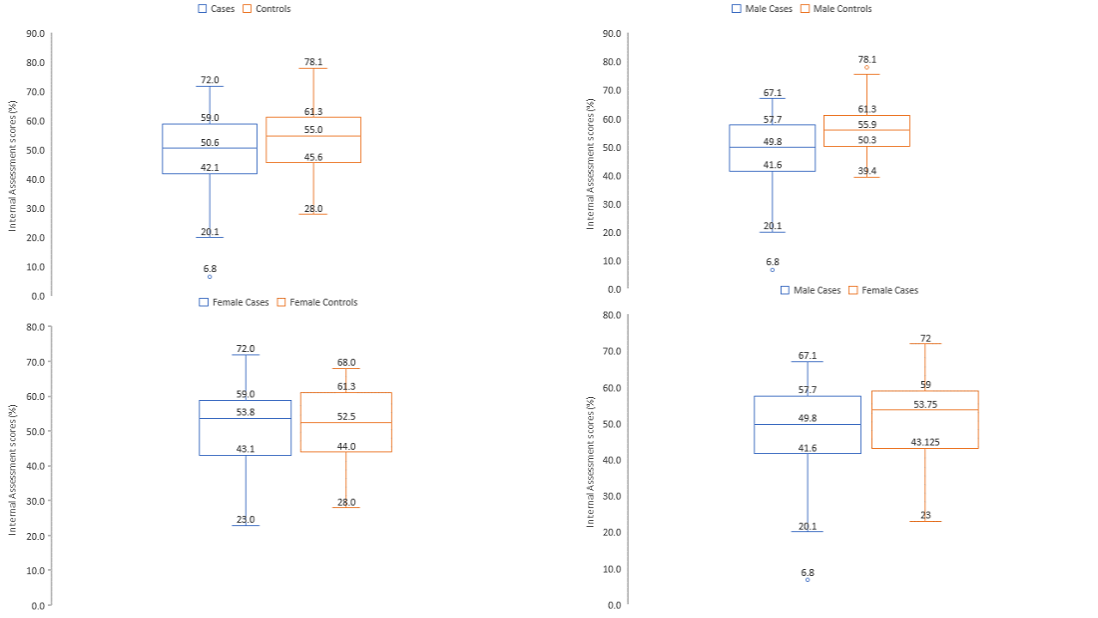
Comparison of mean internal assessment scores across various groups (n=91).

Male cases showed a significantly lower mean score of 9.5% on the first Internal assessment (*P*=.005) and 8.4% on the second internal assessment (*P*=.01) compared to male controls. Female cases observed 0.6% lower scores on both internal assessments than female controls (*P*=.89 and *P*=.59), as shown in Figure 2 and Table 2.

**Table 2:** Comparison of mean international assessment scores between various groups.

Group			First internal assessment scores					Second internal assessment scores		
			Mean (SD)		*P* value		Mean (SD)			*P* value
**Group 1**					.01^a^					.01^a^
	Cases	48.7 (15.0)				50.2 (13.9)				
	Controls	53.9 (12.1)				54.3 (11.5)				
**Group 2**					.05					.83
	Male cases	45.6 (15.5)				50.2 (14.9)				
	Female cases	50.1 (14.0)				50.2 (13.2)				
**Group 3**					.005^a^					.01^a^
	Male cases	45.9 (13.6)				49.3 (14.7)				
	Male controls	55.4 (12.1)				57.7 (10.6)				
**Group 4**					.89					.59
	Female cases	52.2 (13.9)				50.8 (13.3)				
	Female controls	52.8 (12.2)				51.4 (11.5)				

^a^Significant values.

The 7 items of the GAS were analyzed for the 91 cases. A salience score of ≥3 was observed in 30 (33%) participants, 24 (26.4%) had a tolerance score of ≥3, 34 (37.4%) had a mood modification score of ≥3, 20 (22%) had a relapse score of ≥3, 26 (28.6%) a withdrawal score of ≥3, 22 (24.2%) had a conflict score of ≥3, and 56 (61.5%) had a problem score of ≥3 (Multimedia Appendix 1).

Of the 448 students who were surveyed, 28 cases had a GAS score of ≥63. Thus, the prevalence of gaming disorder in this study was 6.3% among the study population and 31% (28/91) among cases.

There was a significant difference observed between mean GAS scores among male and female cases (males: mean 69.5, SD 6.4, n=13 vs females: mean 78.5, SD 9.2, n=15; *P*=.008). The GAS scores of female cases were 9 percentage points higher than male cases.

There was a negative correlation observed between the GAS and mean internal assessment scores for the cases (*r*=–0.02). Further, it was observed that the odds of scoring less than 50% were 1.9 (95% CI 1.04-3.44, *P*=.03) times more among cases than controls. A similar result was observed during the second internal assessment, where the odds of scoring less than 50% were 1.8 (95% CI 0.97-3.36, *P*=.06) times higher among cases than controls.

## Discussion

### Principal Findings

To the best of our knowledge, this study is the first to use a case-control design to examine the association between gaming and scholastic performance in medical students. Since the availability of literature on internet gaming among medical students is sparse, it is difficult to draw meaningful comparisons.

This study observed that smartphones were the most commonly used gaming platform by medical students. The study observed no significant association between the frequency of gaming and internal assessment scores. Gamers (cases) showed a significantly lower score than nongamers (controls). Male gamers showed significantly lower scores compared to male nongamers, whereas the difference between scores of female gamers and nongamers was not statistically significant. The study found a negative correlation between GAS scores and internal assessment scores. Further, there was a higher risk of lower scores among those who played games compared to those who did not.

### Time Spent Gaming and Internal Assessment Scores

There was no significant differences observed in the internal assessment scores and the number of hours spent gaming. This finding differs from a study by Ip et al [9], where frequent gamers (both males and females) scored less than nonfrequent gamers in examinations, with the average grades of nongamers being 9.4% higher than those of frequent gamers. A study conducted by Dumrique and Castillo [10] observed no significant relationships between the number of hours of playing and the social behavior of the respondents. The reason for this difference may be because they included assessments from the whole academic year, whereas we have included only assessments from the last 6 months. In addition, the scale of measurements differs between those studies and our study.

### Internal Assessment Scores and Gaming Among Males and Females

In this study, the mean scores of the first and second midterms of those who played games were 5.2% and 4.1% lower than those who did not play games, respectively. Male nongamers had 9.5% and 8.4% higher scores than male gamers for the first and second assessments, respectively. This is somewhat similar to the finding of Ip et al [9], where the examination grades of infrequent male gamers were on average 7.2% higher compared to regular male gamers. In our study, we observed no significant difference in internal assessment scores between female gamers and female nongamers. We also observed that female gamers had higher internal assessment scores compared to male gamers despite having higher GAS scores. This indicates that although there is a greater incidence of gaming disorder among females, this is not associated with poor scholastic performance. This is similar to the findings of Ip et al [9] on gaming frequency and academic performance, where female students performed better than male students in all disciplines even though they were gaming. Contrary to our finding was Dumrique and Castillo’s [10] observation that the academic performance of students was not affected even if they played online games. This difference is because their participants had better self-control, played games preferably during the weekends, and socialized more. This finding is useful in the context of gaming as a leisure activity that is not done in excess.

### Prevalence of Gaming Disorder Using Various Scales

In this study, gaming addiction, as assessed by the GAS, was found to be prevalent in 6.2% of the study population and 31% of those who played games. The prevalence of gaming disorder using different scales in various prior studies ranged from 2.0% to 22.7% [8,11-23]. This variation may be due to differences in study populations and measurement scales used.

In terms of specific studies, Lemmens et al [8] found the prevalence of the gaming addiction to be 2.3% using the monothetic format and 9.3% using the polythetic format [8]. Mentzoni et al [24], who used the GAS, observed the prevalence of problematic users (score of ≥4 out of 7 on the GAS) to be 4.1%. Wang et al [21] in Hong Kong identified 15.6% of study participants as having a gaming addiction. In a study conducted in Germany by Festl et al [25], 3.7% of the respondents were considered to be problematic gamers.

### Correlation of the GAS With Internal Assessment Scores

We found a negative correlation between GAS scores and mean internal assessment scores—greater gaming disorder scores were associated with lower internal assessment scores, emphasizing the fact that gaming negatively affects scholastic performance. A review by Mihara and Higuchi [26] showed that many studies reported lower grades and career attainment in students indulging in excessive gaming.

Our novel study quantifies the risk of poor scholastic scores associated with excessive gaming, with gamers at higher risk than nongamers (odds ratio 1.9, 95% CI 1.04-3.44). This finding is useful in the context of restricting gaming as a leisure activity than indulging in it excessively. This observation also helps in the early identification and treatment of students who are gaming excessively to prevent poor academic performance.

### Limitations

The study comes with the inherent limitations of the case-control design. The retrospective nature of the study can be used to establish an association between gaming and scholastic performance, but cannot establish causation. Additionally, it should be noted that cases and controls were matched only for age and gender since matching for other potential confounders would have led to overmatching and fewer control participants. Further, the findings of this study pertain to a single educational setting, which could limit its generalizability.

### Conclusion

We conclude that gaming adversely affects scholastic performance among male students compared to female students. Awareness needs to be created among medical students about the negative effects of gaming, which can have a detrimental effect on their scholastic performance. Students, parents, teachers, and institutions should be advised on the early detection of gaming disorder. Treatment services should be made available to those with gaming disorder in medical institutions. The study also opens new avenues for further exploration in different educational settings using a cohort study design to examine the long-term impact of gaming on the scholastic performance of students.

## Appendix

Multimedia Appendix 1 Scoring of items using the Gaming Addiction Scale for cases.

## Abbreviations

GAS: Gaming Addiction Scale

## References

[1] (2020). Gaming Accessories Market - Growth, Trends, and Forecasts (2020-2025). Market Insights Reports.

[2] (2020). Gaming Industry. India Gaming Show.

[3] Spada MM (2014). An overview of problematic internet use. Addict Behav.

[4] (2018). Addictive behaviours: Gaming disorder. World Health Organization.

[5] Ho Roger C, Zhang Melvyn W B, Tsang Tammy Y, Toh Anastasia H, Pan Fang, Lu Yanxia, Cheng Cecilia, Yip Paul S, Lam Lawrence T, Lai Ching-Man, Watanabe Hiroko, Mak Kwok-Kei (2014). The association between internet addiction and psychiatric co-morbidity: a meta-analysis. BMC Psychiatry.

[6] Männikkö N, Billieux J, Kääriäinen M (2015). Problematic digital gaming behavior and its relation to the psychological, social and physical health of Finnish adolescents and young adults. J Behav Addict.

[7] Singh S, Dahiya N, Singh AB, Kumar R, Balhara YPS (2019). Gaming disorder among medical college students from India: Exploring the pattern and correlates. Ind Psychiatry J.

[8] Lemmens JS, Valkenburg PM, Gentile DA (2015). The Internet Gaming Disorder Scale. Psychol Assess.

[9] Ip B, Jacobs G, Watkins A (2008). Gaming frequency and academic performance. AJET.

[10] Dumrique DO, Castillo JG (2018). Online Gaming: Impact on the Academic Performance and Social Behavior of the Students in Polytechnic University of the Philippines Laboratory High School. KSS.

[11] Johansson A, Götestam K Gunnar (2004). Problems with computer games without monetary reward: similarity to pathological gambling. Psychol Rep.

[12] Rehbein F, Kleimann M, Mössle Thomas (2010). Prevalence and risk factors of video game dependency in adolescence: results of a German nationwide survey. Cyberpsychol Behav Soc Netw.

[13] Müller K W, Janikian M, Dreier M, Wölfling K, Beutel ME, Tzavara C, Richardson C, Tsitsika A (2015). Regular gaming behavior and internet gaming disorder in European adolescents: results from a cross-national representative survey of prevalence, predictors, and psychopathological correlates. Eur Child Adolesc Psychiatry.

[14] Rasmussen M, Meilstrup CR, Bendtsen P, Pedersen TP, Nielsen L, Madsen KR, Holstein BE (2015). Perceived problems with computer gaming and Internet use are associated with poorer social relations in adolescence. Int J Public Health.

[15] Rehbein F, Kliem S, Baier D, Mößle Thomas, Petry NM (2015). Prevalence of Internet gaming disorder in German adolescents: diagnostic contribution of the nine DSM-5 criteria in a state-wide representative sample. Addiction.

[16] Vadlin S, Åslund Cecilia, Rehn M, Nilsson KW (2015). Psychometric evaluation of the adolescent and parent versions of the Gaming Addiction Identification Test (GAIT). Scand J Psychol.

[17] Dreier M, Wölfling K, Duven E, Giralt S, Beutel ME, Müller K W (2017). Free-to-play: About addicted Whales, at risk Dolphins and healthy Minnows. Monetarization design and Internet Gaming Disorder. Addict Behav.

[18] Desai RA, Krishnan-Sarin S, Cavallo D, Potenza MN (2010). Video-gaming among high school students: health correlates, gender differences, and problematic gaming. Pediatrics.

[19] Turner NE, Paglia-Boak A, Ballon B, Cheung JTW, Adlaf EM, Henderson J, Chan V, Rehm J, Hamilton H, Mann RE (2012). Prevalence of Problematic Video Gaming among Ontario Adolescents. Int J Ment Health Addiction.

[20] Gentile Douglas A, Choo Hyekyung, Liau Albert, Sim Timothy, Li Dongdong, Fung Daniel, Khoo Angeline (2011). Pathological video game use among youths: a two-year longitudinal study. Pediatrics.

[21] Wang C, Chan CLW, Mak K, Ho S, Wong PWC, Ho RTH (2014). Prevalence and correlates of video and internet gaming addiction among Hong Kong adolescents: a pilot study. Scientific World Journal.

[22] Kim NR, Hwang SS, Choi J, Kim D, Demetrovics Z, Király Orsolya, Nagygyörgy Katalin, Griffiths MD, Hyun SY, Youn HC, Choi S (2016). Characteristics and Psychiatric Symptoms of Internet Gaming Disorder among Adults Using Self-Reported DSM-5 Criteria. Psychiatry Investig.

[23] Thomas NH, Martin FH (2009). Video-arcade game, computer game and Internet activities of Australian students: Participation habits and prevalence of addiction. Australian Journal of Psychology.

[24] Mentzoni Rune Aune, Brunborg Geir Scott, Molde Helge, Myrseth Helga, Skouverøe Knut Joachim Mår, Hetland Jørn, Pallesen Ståle (2011). Problematic video game use: estimated prevalence and associations with mental and physical health. Cyberpsychol Behav Soc Netw.

[25] Festl R, Scharkow M, Quandt T (2013). Problematic computer game use among adolescents, younger and older adults. Addiction.

[26] Mihara S, Higuchi S (2017). Cross-sectional and longitudinal epidemiological studies of Internet gaming disorder: A systematic review of the literature. Psychiatry Clin Neurosci.

