# Supplementary material for: The Association Between Gaming Practices and Scholastic Performance Among Medical Students in India: Case-Control Study
Source: JMIR Med Educ. 2021 Sep 9;7(3):e22235. doi: 10.2196/22235 (PMC8461540; doi:10.2196/22235)
Supplement: Multimedia Appendix 1 [file mededu_v7i3e22235_app1.docx]

**Multimedia Appendix 1.** Scoring of Items using the Gaming Addiction Scale (n= 91).

|  | How often during the last six months… | Never (n,%) | Rarely ((n,%) | Sometimes (n,%) | Often(n,%) | Very often (n,%) |
| --- | --- | --- | --- | --- | --- | --- |
|  | **Items** | 1 (%) | 2 | 3 | 4 | 5 |
| *A* | **Salience** | | | | | |
| *1* | Did you think about playing a game all day long? | 40 ( 44%) | 16 (17.6%) | 16 ( 17.6%) | 13 (14.3%) | 6 (6.6%) |
| *2* | Did you spend much free time on games? | 17 (18.7%) | 23 (25.3 %) | 20 ( 22%) | 21 (23.1%) | 10 (11%) |
| *3* | Have you felt addicted to a game? | 18 (19.8%) | 18 (19.8 %) | 29 (31.9%) | 16 (17.6%) | 10 (11%) |
|  | | | | | | |
| *B* | **Tolerance** | | | | | |
| *1* | Did you spend increasing amounts of time on games? | 25 (27.5%) | 16 (17.6%) | 28  (30.8%) | 16 (17.6%) | 6(6.6%) |
| *2* | Did you play longer than intended? | 20(22%) | 16 (17.6%) | 22 (24.2%) | 25 (27.5%) | 8(8.8%) |
| *3* | Were you unable to stop once you started playing? | 29 (31.9) | 15 (16.5%) | 25 (27.5%) | 13 (14.3%) | 9(9.9%) |
|  | | | | | | |
| *C* | **Mood Modification** | | | | | |
| *1* | Did you play games to forget about real life? | 31 (34.1%) | 15 (16.5%) | 20 (22%) | 18 (19.8%) | 7(7.7%) |
| *2* | Have you played games to release anger or stress? | 26 (28.6%) | 11 (12.1%) | 25 (27.5%) | 20 (22%) | 9 (9.9%) |
| *3* | Have you played games to feel better? | 20 (22%) | 12 (13.2%) | 24 (26.4%) | 25 (27.5%) | 10 (11%) |
|  | | | | | | |
| *D* | **Relapse** | | | | | |
| *1* | Have others unsuccessfully tried to reduce your game use? | 29 (31.9%) | 24 (26.4%) | 16 (17.6%) | 14 (15.4%) | 8(8.8%) |
| *2* | Were you unable to reduce your game time? | 33 (36.3%) | 24 (26.4%) | 21 (23.1%) | 10 (11%) | 3 (3.3%) |
| *3* | Have you failed when trying to reduce the game time? | 34 (37.4%) | 20 (22%) | 17 (18.7%) | 14 (15.4%) | 6 (6.6%) |
|  | | | | | | |
| *E* | **Withdrawal** | | | | | |
| *1* | Have you felt bad when you were unable to play? | 35 (38.5%) | 22 (24.2%) | 13 (14.3%) | 16 (17.6%) | 5 (5.5%) |
| *2* | Have you become angry when unable to play? | 44 (48.4%) | 19 (20.9%) | 14 (15.4%) | 9 (9.9%) | 5 (5.5%) |
| *3* | Have you become stressed when unable to play? | 47 (51.6%) | 16 (17.6% ) | 15 (16.5%) | 10 (11%) | 3 (3.3) |
|  | | | | | | |
| *F* | **Conflict** | | | | | |
| *1* | Did you have fights with others (e.g., family, friends) over your time spent on games? | 42 (46.2%) | 21 (23.1%) | 15 (16.5%) | 10 (11%) | 3 (3.3%) |
| *2* | Have you neglected others (e.g., family, friends) because you were playing games? | 38 (41.8%) | 23 (25.3%) | 18 (19.8%) | 9(9.9%) | 3 (3.3%) |
| *3* | Have you lied about time spent on games? | 35 (38.5%) | 21 (23.1) | 21 (23.1%) | 10 (11%) | 4 (4.4%) |
|  | | | | | | |
| *G* | **Problems** | | | | | |
| *1* | Have you neglected other important activities (e.g., school, work, sports) to play games? | 38 (41.8%) | 19 (20.9) | 17 (18.7%) | 12 (13.2%) | 5 (5.5%) |
| *2* | ...has your time on games caused sleep deprivation? | 34 (37.4%) | 17 (18.7%) | 23 (25..3%) | 9 (9.9%) | 8 (8.8%) |
| *3* | ...did you feel bad after playing for a long time? | 33 (36.3%) | 13 (14.3%) | 24(26.4%) | 14 (15.4%) | 7 (7.7%) |
